# Comprehensive Analysis and Evaluation of Anomalous User Activity in Web Server Logs

**DOI:** 10.3390/s24030746

**Published:** 2024-01-24

**Authors:** Lenka Benova, Ladislav Hudec

**Affiliations:** Faculty of Informatics and Information Technologies, Slovak University of Technology in Bratislava, 842 16 Bratislava, Slovakia; ladislav.hudec@stuba.sk

**Keywords:** anomaly detection, web server, isolation forest, network logs, user behavior, clustering, DBSCAN, expert analysis

## Abstract

In this study, we present a novel machine learning framework for web server anomaly detection that uniquely combines the Isolation Forest algorithm with expert evaluation, focusing on individual user activities within NGINX server logs. Our approach addresses the limitations of traditional methods by effectively isolating and analyzing subtle anomalies in vast datasets. Initially, the Isolation Forest algorithm was applied to extensive NGINX server logs, successfully identifying outlier user behaviors that conventional methods often overlook. We then employed DBSCAN for detailed clustering of these anomalies, categorizing them based on user request times and types. A key innovation of our methodology is the incorporation of post-clustering expert analysis. Cybersecurity professionals evaluated the identified clusters, adding a crucial layer of qualitative assessment. This enabled the accurate distinction between benign and potentially harmful activities, leading to targeted responses such as access restrictions or web server configuration adjustments. Our approach demonstrates a significant advancement in network security, offering a more refined understanding of user behavior. By integrating algorithmic precision with expert insights, we provide a comprehensive and nuanced strategy for enhancing cybersecurity measures. This study not only advances anomaly detection techniques but also emphasizes the critical need for a multifaceted approach in protecting web server infrastructures.

## 1. Introduction

This article builds upon the preliminary research introduced at the 2023 17th International Conference on Telecommunications (ConTEL) [1], with enhancements that include a broader array of case studies and the incorporation of expert evaluations to enrich the understanding and interpretation of the outliers identified through the refined clustering methodology.

In this digital era, the growth of the internet has brought about an increase in security vulnerabilities, making the detection of network intrusions a highly important area of research [2]. A survey [3] reveals that approximately 84% of web applications possess at least one medium-severity vulnerability, while 55% of them contain at least one high-severity vulnerability. The integration of machine learning in cybersecurity represents a pivotal advancement, offering new algorithms that are more effective at spotting and stopping cyber threats. These machine learning applications are now essential in safeguarding network and computer systems against complex attacks.

The heart of network security lies in intrusion detection systems (IDS), which aim to identify unusual activities that might be signs of security breaches. Creating an IDS that can detect new, unknown threats—so-called ‘zero-day’ attacks—is especially challenging but crucial [4].

Traditionally, machine learning for detecting anomalies has focused on overall network traffic, often overlooking the specific behaviors of individual web server users. This gap in analysis is notable because while overall traffic patterns are significant, the distinct actions of users are also key to understanding network anomalies. These details can be lost in the large volumes of data that are generated daily, which can lead to missed security threats if not recognized and managed properly.

Our study addresses this gap by examining the behaviors of individual web server users, aiming to identify anomalies that might be missed when looking only at broader traffic patterns. This detailed focus is essential for an effective IDS, as it allows for the early detection and management of such anomalies, thus improving network security.

**Figure 1:**
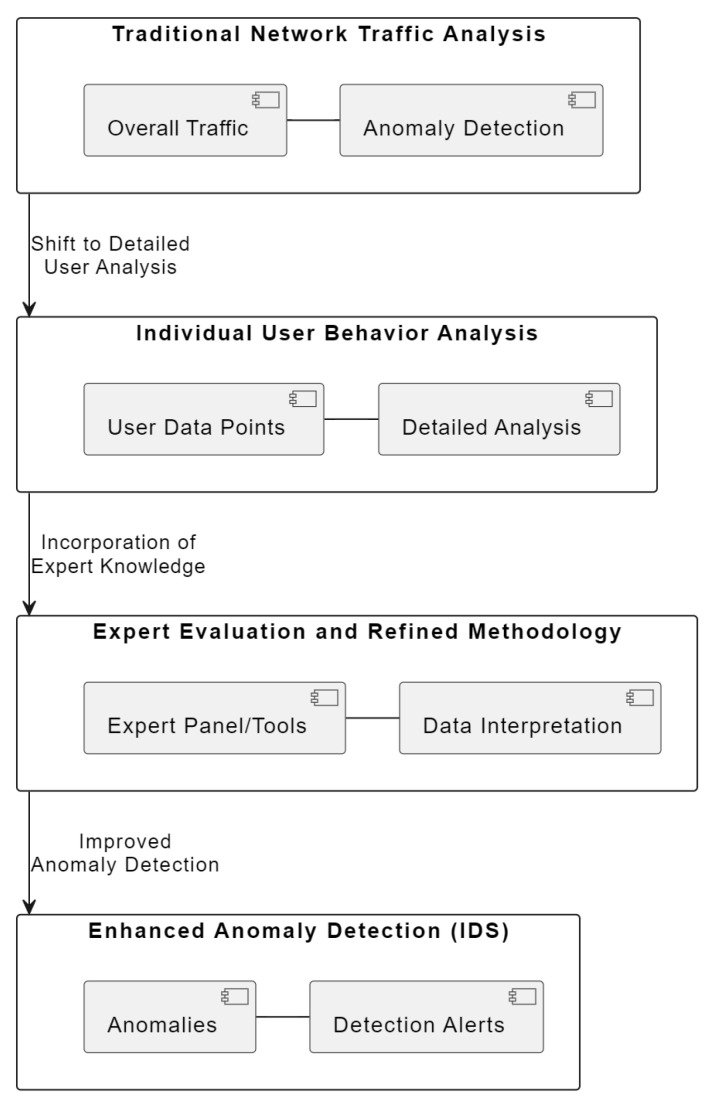
Conceptual layers of network security analysis.

We set out to add depth to the field of anomaly detection by advocating for a closer look at individual user behavior. Our proposal is that a detailed analysis can reveal insights that are not apparent from examining overall network traffic. Figure 1 visually encapsulates this multi-layered approach, emphasizing the transition from a broad view of network traffic to a more granular analysis of individual user actions. This shift is crucial for detecting sophisticated cyber threats that might otherwise go unnoticed. The diagram in Figure 1 illustrates the multi-layered approach adopted in our study for enhancing network security through anomaly detection:

1. Layer 1: Traditional Network Traffic Analysis—represents the conventional method focusing on overall network traffic.
2. Layer 2: Individual User Behavior Analysis—highlights our novel approach of analyzing individual user behaviors.
3. Layer 3: Expert Evaluation and Refined Methodology—depicts the incorporation of expert insights and advanced methodologies.
4. Layer 4: Enhanced Anomaly Detection (IDS)—shows the outcome with the IDS detecting more sophisticated threats.

Each layer builds upon the previous, showcasing our progression from traditional techniques to a more effective anomaly detection system.

In essence, this study introduces a sophisticated approach to anomaly detection, emphasizing the importance of examining individual user behavior. By combining detailed user-level analysis with expert evaluation, we mark a significant step toward creating more sensitive and effective intrusion detection systems. Our findings lay the groundwork for future innovations in network security that are tailored to meet the challenges of our increasingly connected world.

### 1.1. Research Contributions

This section of the paper highlights the key contributions of our research. The primary contributions include the following:

1. Introduction of a Novel Anomaly Detection Approach: We propose a unique methodology focusing on individual user behavior within network traffic, which is a significant deviation from traditional overall traffic analysis.
2. Enhancement of Intrusion Detection Systems: By incorporating expert evaluations and a refined clustering methodology, we enhance the ability of IDS to detect sophisticated and previously unidentified threats, including zero-day attacks.
3. Bridging a Crucial Gap in Network Security Research: Our research addresses a notable gap in the analysis of network traffic anomalies by emphasizing the importance of user-specific behaviors.
4. Foundation for Future Research: The findings and methodologies presented in this study lay the groundwork for future innovations in network security that are tailored to the evolving digital landscape.

### 1.2. Paper Organization

The remainder of the paper is organized as follows:

1. Section 2: We provide a detailed literature review, establishing the context and relevance of our research within the field of network security.
2. Section 3: This section delves into the specifics of our research methodology. It includes the characteristics of the data we used and details our feature engineering approach. It also outlines our advanced anomaly detection methodology, providing the technical foundation of our study.
3. Section 4: We present our findings and interpret the implications of anomalous user behavior.
4. Section 5: This section concludes the paper, summarizing the key findings and limitations.
5. Section 6: We suggest possible future work in the presented field.

## 2. Related Work

Extensive efforts have been dedicated to intrusion detection. Research documented in [5] introduces a blended intrusion detection approach, combining misuse and anomaly detection, utilizing the k-means algorithm. Another study [6] implements the K-nearest neighbor classifier for identifying intrusions. The study in [7] provides a comprehensive overview of machine learning methodologies applied to web intrusion detection. Furthermore, the researchers in [8] suggest a clustering technique aimed at unsupervised anomaly detection.

Numerous methods have emerged for detecting anomalies through the analysis of user behavior. These methods often undergo testing on labeled datasets with a primary focus on clustering or pattern matching. Others employ advanced machine learning techniques to dynamically identify unusual patterns in real-time scenarios.

For the research conducted by Gao, Ma, and Li [9], web logs from the BUPT school websites, which utilized NGINX as a reverse proxy, were collected. The dataset comprised 4877 user sequences, extracted from access logs spanning five days in 2017, specifically from March 16 to March 20. The authors further acquired 52,713 attack logs from the MACCDC2012 dataset, which were analyzed to assess detection and false alarm rates. Utilizing the extracted attributes, the authors scrutinized user behaviors and web server log data. They identified four critical characteristics from the logs and developed a user access sequence to simulate user behavior. The authors applied two algorithms for detecting network threats based on user behavior, one involving entropy calculations for user sequences and the other employing machine learning to cluster diverse user behaviors. The results showed a detection rate of 100% and a false alarm rate of 1.68% when the distance fell between 1.5 and 1.6, indicating the successful identification of all 12 fraudulent users. Similarly, for distances between 1.7 and 2.0, a false alarm rate of 1.33% was achieved with a detection rate of 96.67%, signifying the detection of 11 out of 12 fraudulent users. Unlike the approach by Gao, Ma, and Li, which focused on entropy calculations and machine learning clustering, our method employs Isolation Forests to efficiently handle larger datasets and isolate anomalies based on unique user behaviors, offering a more scalable solution for large-scale web server log analysis.

Debnath et al. [10] introduced LogLens, an automated real-time log analysis system designed to identify anomalies with minimal human intervention. The system categorizes anomaly detection methods into two groups: stateless and stateful. Notably, LogLens effectively detected spoofing attacks in Signaling System No. 7 (SS7) logs using a log sequence anomaly detector. In contrast to LogLens’ categorization of anomaly detection into stateless and stateful methods, our approach introduces a nuanced analysis of user behaviors through detailed feature engineering, providing a deeper insight into user interactions and potential security threats.

Zhao et al. [11] conducted a study to evaluate the performance of five widely employed unsupervised log anomaly detection algorithms. Their investigation aimed to address two key research questions:

1. How effectively do these approaches perform on various real-world log data containing diverse anomalous patterns?
2. What is the overall effectiveness of these techniques?

The analyzed algorithms encompassed statistical modeling methods, such as PCA [12], LogCluster [13], and IM [14], as well as deep learning-based approaches, including DeepLog [15] and LogAnomaly [16]. To assess their effectiveness, ten datasets, comprising various log types like application errors, user operations, and system logs, were prepared. The evaluation criteria included precision, recall, and micro-F1-score metrics.

The results demonstrated that deep learning-based methods consistently outperformed conventional statistical methods in detecting anomalies in real-world data. However, it was noted that the performance of these methods varied across different datasets. The average micro-F1-score across all datasets was 0.48066, as detailed in Table 1.

Based on their findings, the authors proposed LogAD, which is a comprehensive log anomaly detection system designed to address these challenges. LogAD integrates a knowledge base derived from domain expertise, visually interpretable reporting, and a combination of various anomaly detection algorithms to handle a wide array of abnormal patterns. The system employs ensemble learning principles, allowing engineers to select suitable anomaly detection components based on their domain knowledge to identify specific anomalous patterns within different logs. While prior work utilized multiple methods (including PCA, LogCluster, and IM) for more accurate anomaly identification, it lacked the versatility to handle a broad spectrum of anomalous patterns. Notably, the authors introduced a multivariate time-series anomaly detection method for template count, based on LSTM [17], to identify anomalies using the widely adopted distribution distance measurement (JS divergence). The template sequence and variable value techniques were refined based on earlier research, resulting in an average F1-score of 0.83, confirming the effectiveness of LogAD.

Additionally, LogMine [18], equipped with a clustering module, rapidly clusters large batches of input logs under stringent constraints. The pattern recognition module developed by the authors generates distinct patterns for each cluster, and these patterns form the leaf level of the pattern hierarchy. Through iterative processes that gradually relax constraints and merge clusters to create more generalized patterns, LogMine constructs a hierarchical pattern structure. This process continues until the most general pattern is achieved or the pattern hierarchy is fully constructed. Notably, LogMine is the first framework of its kind, offering unsupervised, scalable, and heterogeneity-resistant log analysis. On distributed platforms, LogMine can process millions of logs within seconds thanks to its one-pass architecture and minimal memory footprint. The framework demonstrates scalability to handle hundreds of millions of logs effectively.

While Zhao et al. assessed various unsupervised log anomaly detection algorithms, our method integrates expert evaluations for validation, ensuring not only statistical relevance but also practical applicability in detecting network security anomalies. Building on the limitations identified in LogAD’s ability to handle diverse anomalous patterns, our methodology adopts a more flexible approach through the use of Isolation Forests, which allows for the detection of a broader spectrum of anomalies without requiring extensive domain-specific knowledge. In summary, our research contributes to the field of anomaly detection by offering a scalable, efficient, and versatile approach. Our method’s ability to process extensive datasets with high precision, coupled with a comprehensive validation process, sets it apart from existing methods, addressing key challenges in network security anomaly detection.

## 3. Materials and Methods

### 3.1. Data Characteristics and Feature Engineering

The research leverages an extensive dataset provided by ESET, which is a cybersecurity company based in Slovakia. ESET specializes in cybersecurity solutions and operates infrastructure that enables the automatic updating of virus signatures and software modules on client systems. These updates are delivered from ESET’s secure update servers, which are meticulously monitored for reliability and security. The monitoring process is facilitated through the Zabbix system, which is an open-source monitoring solution widely used in the IT industry. Zabbix logs and tracks update requests, providing valuable insights into the performance and security of the update server infrastructure. These update requests are handled by the NGINX web server, which is a high-performance web server known for its efficiency and scalability. The NGINX web server logs capture critical information about the incoming requests and server responses. This comprehensive dataset forms the basis for detecting anomalies, which may indicate cyber threats such as targeted attacks or unusual behavior patterns within the network.

The dataset comprises NGINX web server logs, reflecting detailed client–server interactions during update distributions. To maintain user privacy in compliance with GDPR, personal identifiers like IP addresses are omitted. Instead, unique hardware fingerprints and update timing requests are logged. The dataset is vast, with 15 GB of compressed logs, which equates to an extensive volume of data to be analyzed.

Feature extraction from the dataset was performed with a parsing tool that uses the hardware fingerprints and time of update requests to distinguish users. Prior research in log analysis [12,14,19,20] has excluded timestamp values from log entries, focusing exclusively on log keys for identifying anomalies. However, in the context of web server user anomaly detection, incorporating timestamps becomes crucial. This approach allows for a more nuanced analysis of user behavior patterns over time, providing key insights into anomalies that might be time-dependent or related to specific usage periods. Unlike previous methodologies that omitted timestamp data, our method leverages this temporal dimension to enhance the accuracy and depth of anomaly detection. Additionally, in our research, the http_user_agent field was parsed to enumerate the types of HTTP requests and response codes, providing insight into client system specifications and language preferences. The connection between the user’s hardware fingerprint and the server access timings is showcased in Table 2, which includes a timeline of requests made by the user during specified periods.

The choice of features such as hardware fingerprints and update timing requests is pivotal to our anomaly detection framework. Hardware fingerprints provide a unique identifier for each user, enabling us to track individual behavior patterns without compromising user privacy. The timing of update requests is crucial for identifying unusual patterns that deviate from the norm, which might indicate potential security threats. The parsing of the http_user_agent field helps us understand the diversity in client systems, which is essential for distinguishing between normal variability in user behavior and genuine anomalies. These features provide a multidimensional view of user interactions with the server, which is crucial for effectively isolating anomalous patterns from typical usage trends.

The naming of features is based on 30-min intervals throughout a day with each interval numbered sequentially. For instance, feature ‘3’ would account for requests within the third 30-min segment of the day (from the 60th to the 90th minute). The entire day is divided into 48 such segments, reflecting the comprehensive scope of data collection.

**Figure 2:**
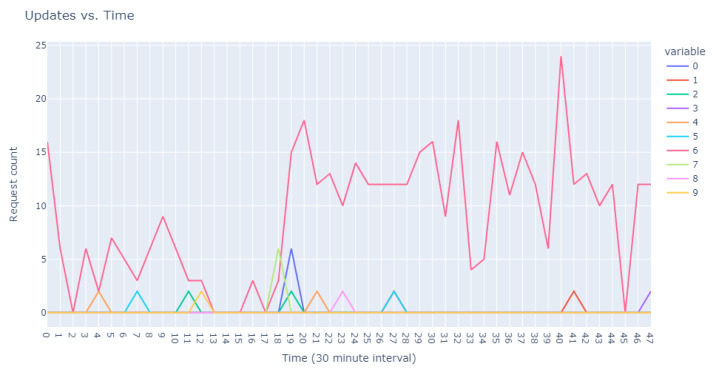
User request time plot.

This dataset is intentionally unlabeled; the unprecedented nature of user-based anomaly investigation on this dataset means that no pre-existing labels can classify request sequences as anomalous. Instead, anomaly detection techniques are employed to autonomously detect these anomalies. The variability of user request behavior is exemplified in Figure 2, where even a mere glance at the activity of a small sample of users reveals the diverse nature of their behavior. Expert analysis of this behavior across the full dataset would be incredibly time consuming.

With over 8.48 million distinct users engaging with a single web server in Europe in a single day, the dataset provides a robust platform for analyzing user behavior and identifying anomalies. This study aims to harness these data to develop an anomaly detection framework that can operate effectively at this scale, ensuring robust network security in an era where the volume and sophistication of cyber threats are continuously escalating.

### 3.2. Advanced Anomaly Detection Methodology

Our approach to identifying anomalies within web server data leverages the cutting-edge capabilities of Isolation Forests, which is an algorithm that sidesteps the traditional, often computationally intensive methods of anomaly detection. Traditional models typically require the measurement of distance or density between data points—a process that can be prohibitively slow with large datasets. Isolation Forests, by contrast, are particularly adept at handling large volumes of data like ours, obtained from extensive web server logs, due to their efficient use of system resources [21]. This makes the algorithm particularly suitable for our dataset, where anomalous patterns are not defined by proximity to normal instances but by their divergence in behavior. The algorithmic efficiency of Isolation Forests is particularly noteworthy when compared to traditional methods like DBSCAN or SVM. Isolation Forests require significantly less computational power and memory, which is especially beneficial for our large dataset of 8.48 million users. This efficiency stems from the algorithm’s use of a random forest approach to isolate anomalies, which is less computationally intensive than calculating distances or densities between every pair of points.

Initially, we considered the application of DBSCAN for clustering user behaviors. However, we faced considerable challenges related to computational efficiency, being restricted to clustering subsets of 100,000 users from our dataset with a memory limitation of 64 GB. Given these constraints, we transitioned to utilizing Isolation Forests, which circumvent the need to model the ‘normal’ data, focusing instead on isolating anomalies and thereby significantly reducing memory overhead.

Isolation Forests operate on two principal characteristics of anomalous data: they are few in number compared to normal instances, and they have attribute values that significantly diverge from those of normal instances. The Isolation Forest algorithm iteratively separates data in the feature space using a binary tree structure, known as an iTree, isolating anomalies closer to the root of the tree due to their rarity and distinctness [21].

Our methodology comprises two distinct approaches for anomaly detection in the dataset [1]. Initially, our first model inspected an extensive dataset comprising over 8.4 million records and 57 features—including HTTP methods, status codes, and update request frequencies within 30-min intervals throughout a day. In configuring the Isolation Forest, we set the number of trees to 100 and used a sub-sampling size equal to the square root of the dataset length, as per the standard practice. This setup balances computational efficiency with the accuracy of anomaly detection. The choice of these parameters was influenced by preliminary tests, which indicated an optimal balance between the model’s sensitivity to anomalies and its ability to handle the vast data volume without significant memory overhead. The Isolation Forest model successfully identified approximately 60,000 anomalies. We followed this with a comprehensive statistical analysis to delineate the data’s distribution, central tendencies, and variability, as detailed in Table 3. Such an analysis is pivotal in discerning whether the anomalies represent isolated instances or indicative of wider trends, thereby contributing to a more nuanced understanding of the network’s security landscape (Table 4).

**Figure 3:**
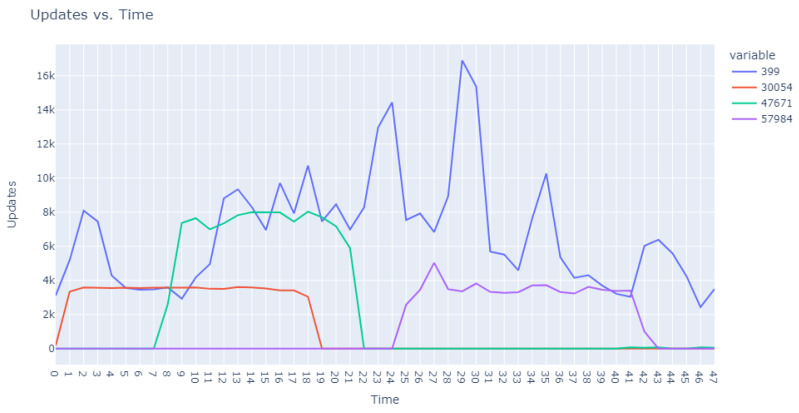
Anomalous user’s request behavior.

**Figure 4:**
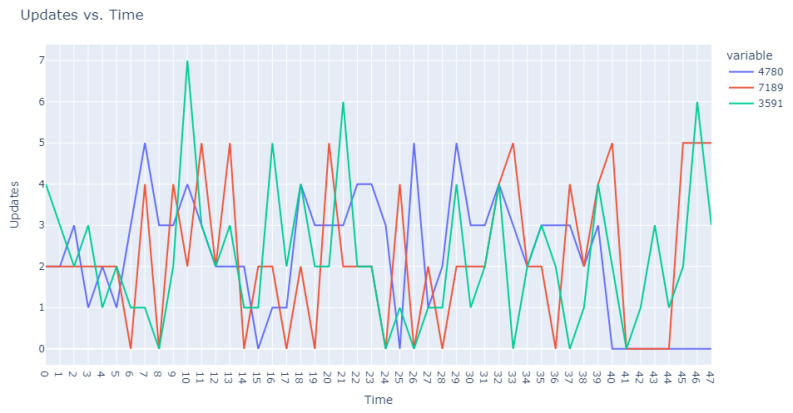
Anomalous user request sequences.

The second facet of our method focused explicitly on the sequences of user requests. Adapting our dataset to include ratios of requests in specific intervals against total user requests, we configured an Isolation Forest model equipped with 48 features representing the timing of user requests and set to 100 estimators. This model identified 18,000 anomalous sequences from a single day’s data, using a contamination factor of 0.05 to isolate the most anomalous top 0.5%. These sequences were then ranked based on anomaly scores provided by the model, which are catalogued in Table 5. Visual representations in Figure 3 and Figure 4 illustrate that the identified anomalies differed between the two applied models, showcasing the multidimensional capability of our anomaly detection methodology.

The adoption of Isolation Forests, complemented by detailed statistical analysis, forms the cornerstone of our anomaly detection framework. This approach aligns with our objective to develop a robust and scalable solution capable of identifying subtle and complex anomalies in large-scale web server data, which is a crucial step in advancing network security in the current digital era.

## 4. Enhanced Interpretation of Anomalous User Behavior

In the quest to unravel the complexities of user interactions with web servers, it is imperative to scrutinize the anomalies that deviate from normal behavior. The use of advanced analytical techniques, such as the Isolation Forest algorithm, has shed light on these irregularities. This section provides a comprehensive account of the methods we implemented to visualize and interpret these anomalies through clustering, emphasizing the synergy between visualization and pattern recognition in the analysis of data anomalies.

### 4.1. Visualization and Initial Analysis of Anomalies

The first phase in our analytical journey was to capture the essence of the most striking anomalies through visualization. We curated a subset of the 1000 most peculiar sequences as identified by the Isolation Forest. These sequences were illustrated using line plots, which served as our primary tool for visual exploration, pinpointing unusual patterns in update request sequences.

**Figure 5:**
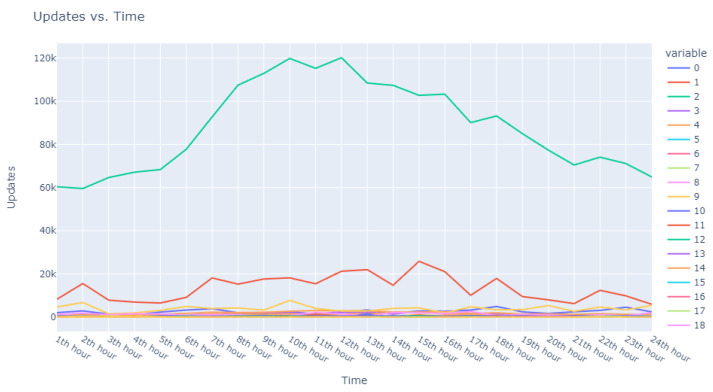
Broad overview of user request behavior.

The visualization commenced with an expansive view, as portrayed in Figure 5. This overview was instrumental in revealing divergent update download sequences that marked the beginning of our inquiry into the anomalies.

**Figure 6:**
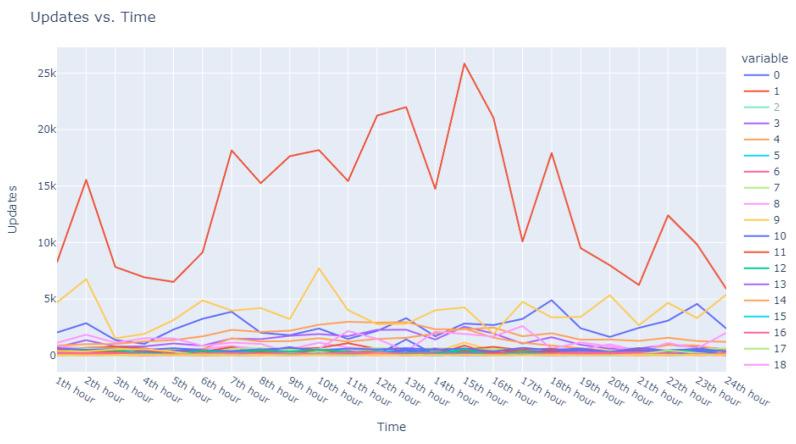
Enhanced view of user request behavior.

**Figure 7:**
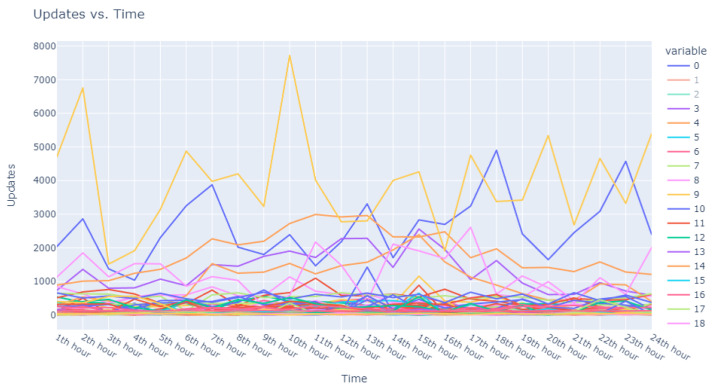
Fine-grained analysis of user request anomalies.

Progressing from the general to the specific, we intensified our focus on the extremities within the data. Figure 6 and Figure 7 present successively refined views, allowing us to dissect and understand the subtleties of the anomalous patterns with increasing precision.

### 4.2. Advanced Clustering for Pattern Recognition

Upon the completion of our initial visual analysis, we transitioned to clustering to dissect the intricacies of the data. The aim was to identify patterns and segregate outliers, which were pivotal in understanding the structure and distribution of anomalies within the vast dataset.

#### 4.2.1. Preparation and Clustering Methodology

The foundation of effective clustering lies in the judicious selection and preparation of data points. By leveraging the Isolation Forest’s identification of the top 1000 anomalies, we ensured a focus on the most significant deviations. Scaling the data with StandardScaler was a preparatory step that allowed for an equitable comparison across different metrics, thereby setting the stage for accurate clustering.

Adopting DBSCAN for our clustering needs was a strategic decision. It is a model resilient to noise and adaptable to the uneven density of real traffic data, which was crucial for the authenticity of our analysis.

##### Optimization of DBSCAN Parameters

The optimization of DBSCAN’s parameters was approached with a data-driven mindset. We ascertained that setting min_pts to double the number of features (96 for our 48 features) and employing the elbow method for the eps value was the most efficient strategy. This choice was vindicated by the emergence of clearly defined clusters, which were rich in information and instrumental for further exploration.

#### 4.2.2. Visualising Clustered Data

**Figure 8:**
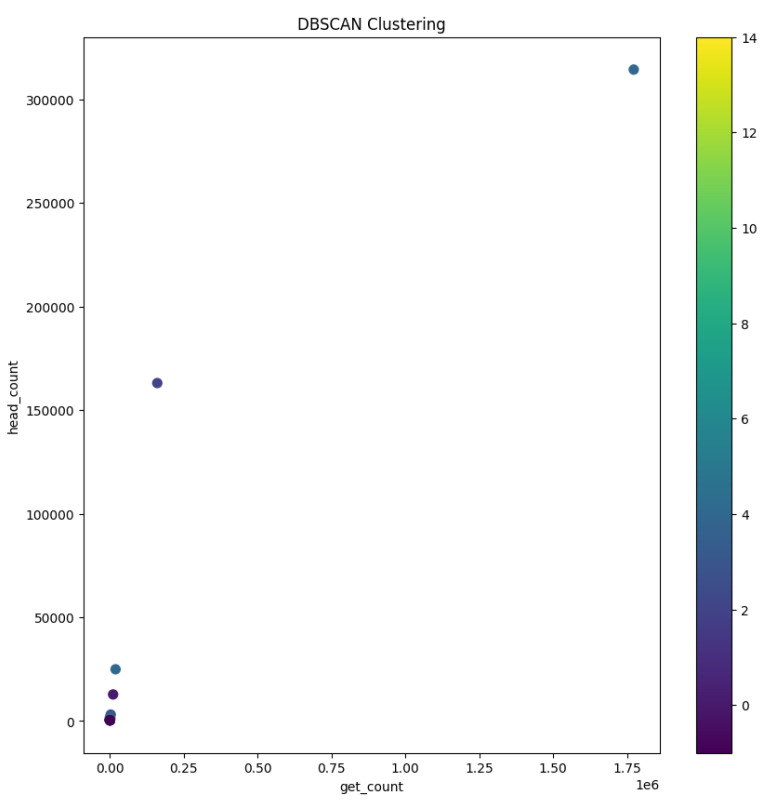
Visual representation of DBSCAN clustering: GET and HEAD request counts.

The visualization of the clustered data is a compelling narrative of user behavior. As demonstrated in Figure 8, distinct clusters are depicted in varying colors, where each color demarcates a group of users with similar GET and HEAD request patterns.

This clustering not only reveals groupings of user behaviors but also poses questions about the underlying reasons for such clustering, suggesting avenues for further investigative research.

Through the meticulous process of visualization followed by the analytical depth provided by DBSCAN clustering, we have taken significant strides in deciphering the complex tapestry of user behavior within our web server logs. This dual approach has laid the groundwork for developing more nuanced and informed strategies to address these anomalies.

### 4.3. Comprehensive Analysis through Dimensionality Reduction

Conducting a comprehensive analysis necessitated addressing the high-dimensional nature of our dataset. To address this challenge, we employed the advanced dimensionality reduction technique known as t-Distributed Stochastic Neighbor Embedding (t-SNE), which is a method introduced by Hinton and Roweis in their work [22]. This unsupervised, non-linear approach is specifically tailored to facilitate the exploration and visualization of data residing in high-dimensional spaces.

At its core, t-SNE excels in preserving the local structure and relative proximities of data points, faithfully representing their intricate high-dimensional relationships in a more accessible two-dimensional map. Consequently, it emerges as a vital tool for unearthing latent data structures within a framework conducive to human interpretation.

Following the application of the DBSCAN clustering algorithm, we leveraged t-SNE in conjunction with the interactive visualization capabilities offered by Plotly to delve deeper into the clustering outcomes. Using the same dataset employed for DBSCAN clustering, which was now augmented with cluster labels, we engaged t-SNE to perform dimensionality reduction. The resulting components, representing the condensed dimensions, were seamlessly integrated into our dataset, enriching it with a new layer of insightful information.

**Figure 9:**
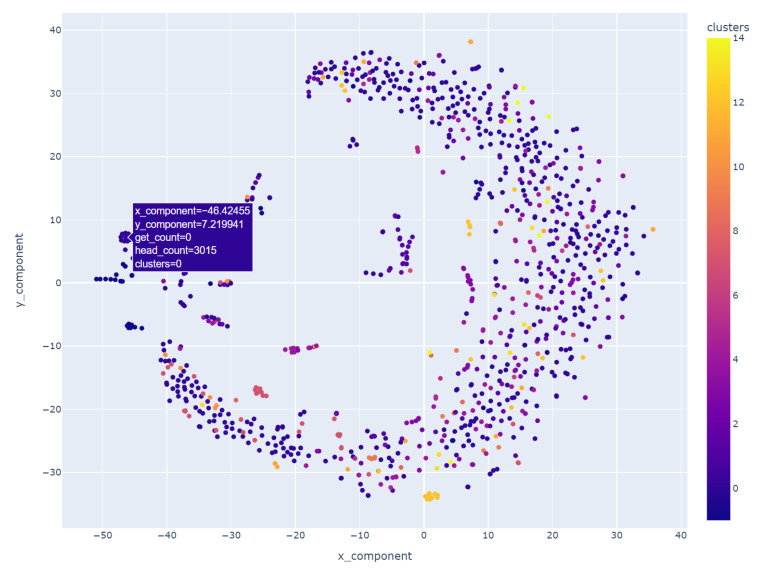
Visualizing clusters post t-SNE application.

The visual representation, depicted in Figure 9, employs a spectrum of colors to differentiate the numerous clusters. Each distinct hue signifies a unique cluster formed by the inherent patterns in user requests. This vibrant visualization functions as a navigational aid, guiding us through the diverse data regions delineated by the collaborative efforts of DBSCAN and t-SNE.

**Figure 10:**
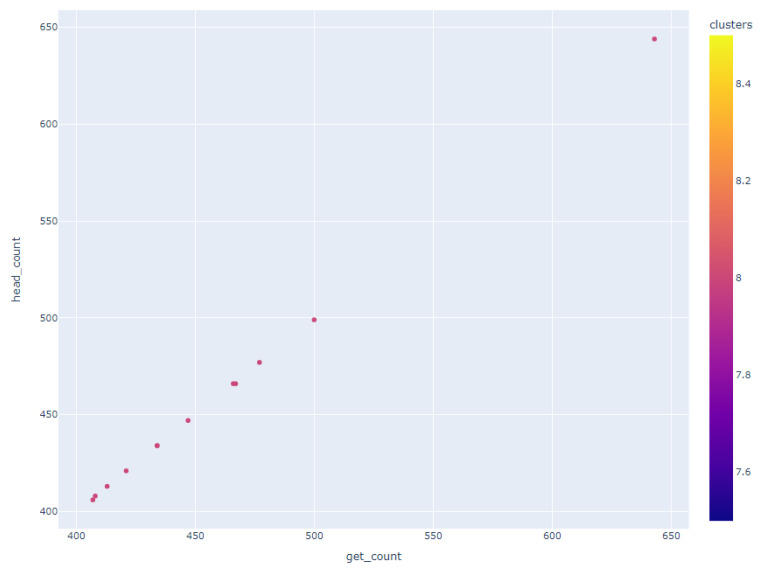
Cluster demonstration of proportional GET and HEAD requests.

Upon closer examination of Figure 9, distinctive clusters became apparent. Notably, one such cluster, illustrated in Figure 10, highlighted a subset of users whose GET and HEAD request volumes displayed either perfect alignment or minor deviations, which was suggestive of a linear correlation between these data points.

Geographical clustering emerged as a prominent pattern with users hailing from specific countries, notably Spain, Russia, Poland, France, and Italy, forming distinct clusters. This observation is of particular significance as it suggests that users from Slovakia, the Czech Republic, Germany, and Hungary share a common characteristic—a similar frequency in GET and HEAD requests—setting them apart from the behavior exhibited by users from other countries.

In stark contrast, another cluster brought to our attention individuals who, irrespective of their global locations, initiated an exceptionally high number of HEAD requests compared to GET requests. This behavior transcended geographical boundaries, but one outlier within this cluster drew notable attention: a user without a specified country of origin who generated an astonishing 1.77 million GET requests alongside approximately 314,000 HEAD requests. This user, along with others exhibiting a substantial disparity between GET and HEAD request counts, constituted a cluster that exemplified the extremes of user behavior within our dataset.

These profound insights, meticulously distilled through the application of t-SNE, amplified by the visual capabilities of Plotly, and interpreted within the context of DBSCAN clustering, have bestowed upon us a nuanced comprehension of user behaviors. Such a nuanced understanding holds immense value as it informs targeted strategies for user engagement, cybersecurity, and resource optimization within the digital landscape we navigate.

### 4.4. Enhanced Clustering of User Request Timings

In our subsequent analysis, we narrowed our focus to the temporal patterns of user requests throughout the day, deliberately excluding other attributes to ensure a pure examination of temporal data. This approach was chosen to maintain the integrity of our analysis, preventing potential skewing of results by high request counts, which had already been explored through previous methods, including the Isolation Forest and our initial clustering approach. Instead, we opted for a relative perspective, evaluating the proportion of a user’s requests within specific 30-min intervals. This strategic choice was made to discern and comprehend anomalies in user request patterns without the undue influence of absolute request quantities.

In pursuit of this nuanced anomaly detection, we initially employed the elbow method to identify potential anomalous clusters within our dataset. Adhering to a conventional heuristic, we set the min_pts parameter at twice the number of features, resulting in a value of 96. However, this approach did not yield the expected insights for our dataset, primarily leading to the identification of a single cluster amid scattered noise—contrary to our objective of discovering multiple anomalous patterns.

**Figure 11:**
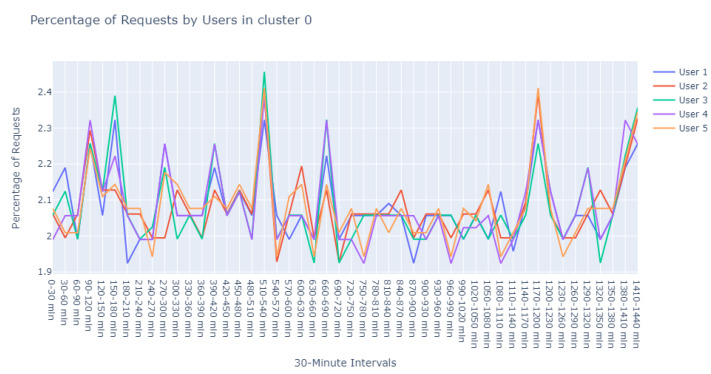
DBSCAN with min_pts set to 3, illustrating cluster 0.

Undeterred, we pursued an iterative approach with the DBSCAN algorithm, experimenting with various combinations of min_pts and eps values, specifically on the user request times data (features 0–47). These iterations consistently resulted in one or two primary clusters: a dominant cluster containing predominantly noisy samples and another encompassing the majority of the remaining data. Notably, an intriguing configuration emerged when setting min_pts to 3, leading to the emergence of a small cluster comprising 5 users, as visualized in Figure 11, with the remainder categorized as noise.

Adjusting the min_pts parameter to 4 yielded outcomes closely resembling those obtained with the 3 min_pts setting. Conversely, a min_pts value of 1 resulted in an excessively fine-grained clustering with each data point forming its own cluster. Through a systematic process of elimination, we determined that setting min_pts to 2 was the optimal choice for our objectives.

**Figure 12:**
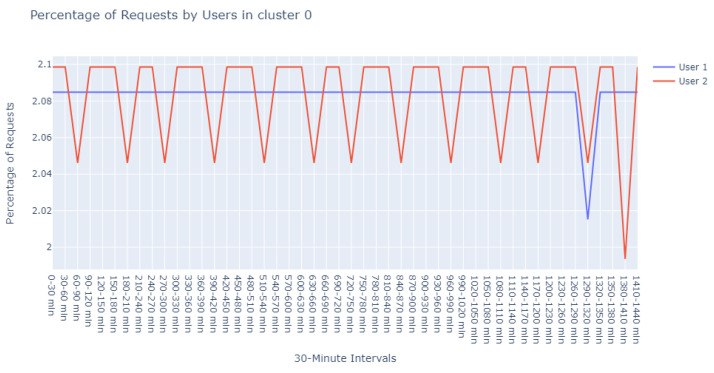
Visualization of cluster 0 with min_pts set to 2 in DBSCAN.

**Figure 13:**
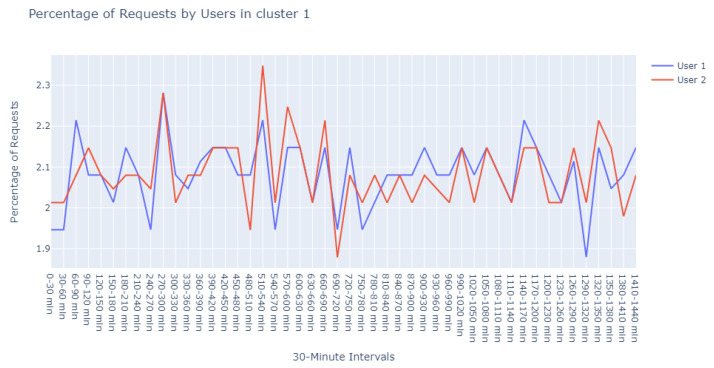
Visualization of cluster 1 with min_pts set to 2 in DBSCAN.

With a min_pts setting of 2, we identified 991 points as noise, 2 points within cluster 0 (refer to Figure 12), another 2 within cluster 1 (refer to Figure 13), and intriguingly, the same 5 points in cluster 2 that were previously observed with the 3 min_pts configuration.

**Figure 14:**
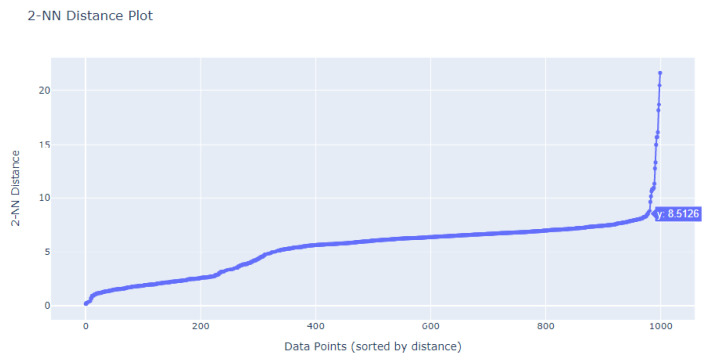
Determining the eps value for DBSCAN using k-NN.

Our exploration of the appropriate eps value for the DBSCAN algorithm involved employing the K-Nearest Neighbors (K-NN) Algorithm. We closely examined the corresponding k-distance graph to identify the “elbow” point—the juncture where the graph’s ascent exhibits a marked steepening. The eps value that corresponds to this elbow point is typically a strong candidate for DBSCAN. In our dataset, this pivotal elbow point was identified at a y-value of 8.5, as illustrated in Figure 14.

**Figure 15:**
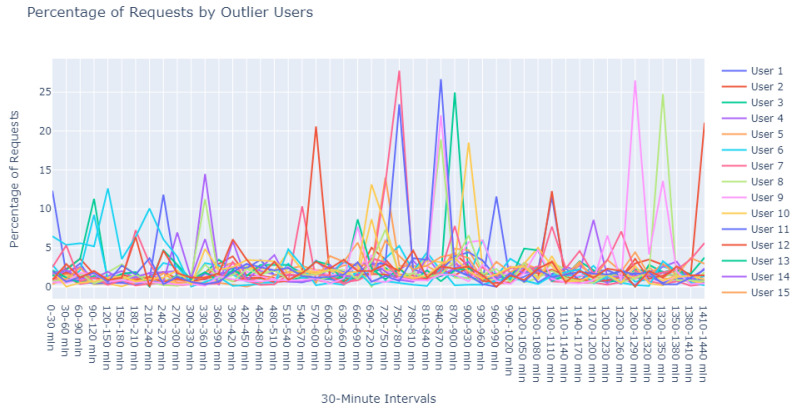
Isolated noise samples using an eps of 8.5 and min_pts of 2 in DBSCAN. Additional noise points are depicted in detail in Figure 16, Figure 17, Figure 18, Figure 19 and Figure 20.

Employing this eps value in conjunction with a min_pts setting of 2, we identified a prominent cluster consisting of 978 data points, while 22 outliers were designated as noise. These 22 points, depicted in Figure 15, were earmarked for further scrutiny to augment our subsequent research efforts.

The results obtained from this clustering analysis, when evaluated in conjunction with the insights from our Isolation Forest model, have elucidated distinct correlations among various user entities deemed anomalous. These aggregated data represent a valuable source for cybersecurity experts, providing them with substantive, actionable insights that can be efficiently assimilated into existing network monitoring frameworks. The expedited detection and subsequent reaction to these atypical patterns underscore a considerable advancement in reinforcing and accelerating the efficacy of cybersecurity protocols. Such enhancements are pivotal in maintaining the integrity of network security and in the preemptive mitigation of potential threats. Integrating the insights from this clustering example into cybersecurity strategies amplifies our defensive capabilities in several ways:

1. Rapid Detection and Response: Our findings enable the development of a cybersecurity framework that promises expeditious detection and immediate response to anomalous activities. This swift action is critical in preempting potential cyber threats and reinforcing network defenses.
2. Predictive Analytics Enhancement: The research contributes to the predictive analytics domain of cybersecurity. By understanding the correlation between user behavior patterns and anomalies, we can forecast potential security incidents and proactively put safeguards in place.
3. Resource Optimization: The precise nature of our analysis allows for an optimized allocation of cybersecurity resources, directing focus and investment toward the most vulnerable points in the network and ensuring a robust defense where it is needed most.
4. Automation of Threat Detection: Leveraging the patterns identified through this research can lead to a higher degree of automation in threat detection. This reduces the need for constant human monitoring and accelerates the activation of defensive protocols.
5. Security Measure Refinement: The data-driven approach of our clustering method enables the fine-tuning of existing security infrastructures, such as firewalls and intrusion detection systems, making them more adept at combating the specific types of anomalies identified.
6. Customized Security Policies: Informed by the unique patterns and clusters of user behavior, our research allows for the creation of tailored security policies. These policies can more effectively address the distinct security challenges presented by different user groups or network segments.
7. Tool Intelligence Augmentation: Feeding the insights derived from our clustering into cybersecurity tools can substantially enhance their intelligence. This improvement makes these tools more capable of identifying, analyzing, and neutralizing sophisticated cyber threats.

By applying these insights, cybersecurity professionals can significantly amplify the effectiveness of the protocols designed to safeguard digital infrastructures. The clustering of anomalous user request patterns thus becomes a powerful instrument not only for immediate threat mitigation but also for the strategic shaping of a more resilient cybersecurity posture.

### 4.5. In-Depth Expert Analysis

The validation of our anomaly detection model was conducted through a rigorous process involving expert evaluations. These evaluations were critical in assessing the practical significance of the identified anomalies. Expert in cybersecurity analyzed the flagged anomalies to determine their relevance and potential impact on network security, ensuring that our model’s findings are not just statistically significant but also practically relevant.

The detailed evaluation of the specialist has been carefully documented, which encompasses the following assessments:

1. Assessment of Figure 11:
  The domain expert provided a reassuring evaluation of the patterns illustrated in this cluster. According to their expertise:
  “The user behavior depicted in this cluster seems to align with standard operational procedures. It’s reasonable to infer that these are end-users engaging with the system during peak hours, likely to obtain updates or patches. The variation in data volume can be attributed to the specific nature of the updates, which may differ based on the product version and type. Minor discrepancies in the data are expected and do not immediately suggest abnormal activity. On the whole, the representation is indicative of a healthy system with user engagement that corresponds with the designed functionality of the update process.”
2. Insights on Figure 12:
  Delving deeper into the nuances of this cluster, the expert offered a nuanced perspective:
  “Upon examining User1 within this cluster, the periodicity of requests is peculiar—deviating from the common patterns observed in typical user behavior. This could potentially point to automated scripts or bots, which operate on a schedule not tied to human activity patterns. Conversely, it might represent a mirroring service designed to replicate data across servers.
  For User2, the observed consistency in request percentages, devoid of significant variance, elicits concern. This pattern may suggest an account that routinely attempts to access the server, likely with invalid credentials. Such persistent yet unfruitful attempts could be symptomatic of either misconfigured legitimate services or probing by unauthorized entities.”
3. Review of Figure 13:
  In this instance, the expert’s appraisal emphasized a potential linkage to update cycles:
  “There’s a discernible synchronicity in the request patterns of these users, suggesting a probable interrelation with the timing of update releases. The simultaneous increase and decrease in activities across users indicate a collective behavior typically associated with the distribution of updates. Considering the users’ activities reflect changes at similar intervals, it’s plausible to deduce that they are responding to the same stimuli—most likely the availability of new updates. This simultaneity may also allude to the handling of different product versions, which are often updated in unison, leading to a concurrent pattern of user engagement.”

In synthesizing the expert’s analysis, it becomes apparent that while typical user behaviors, such as downloading updates at scheduled times, represent the expected operational flow, there are several outliers that warrant further investigation. Particularly, the presence of anomalies such as potential unauthorized bots or users consistently failing to authenticate poses security questions. Moreover, the synchronized behavior in relation to update releases could be shedding light on systematic user interactions with our products, indicative of both standard and coordinated activities, possibly influenced by the rollout of multi-version product updates. Such insights are invaluable for refining user behavior models and predicting potential system abuses or technical issues, contributing to enhanced system integrity and proactive security measures.

**Figure 16:**
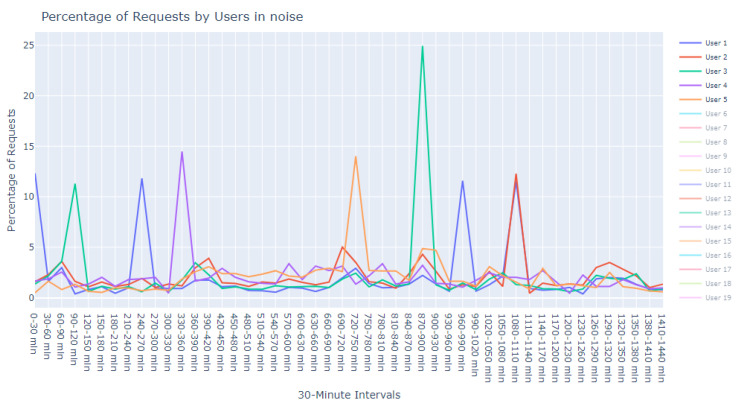
Anomalous noise users, user 1 to 5.

**Figure 17:**
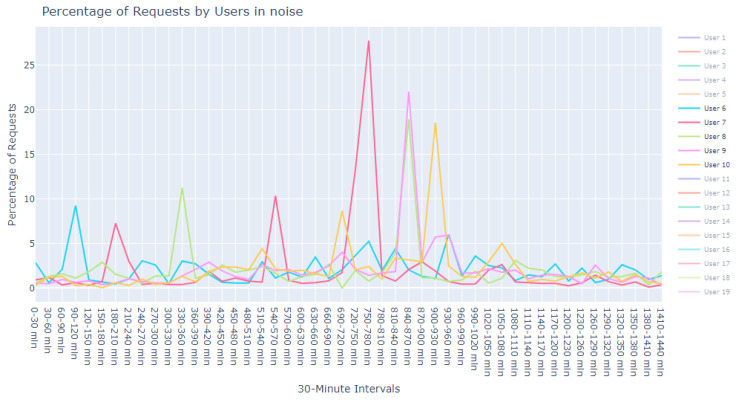
Anomalous noise users, user 6 to 10.

**Figure 18:**
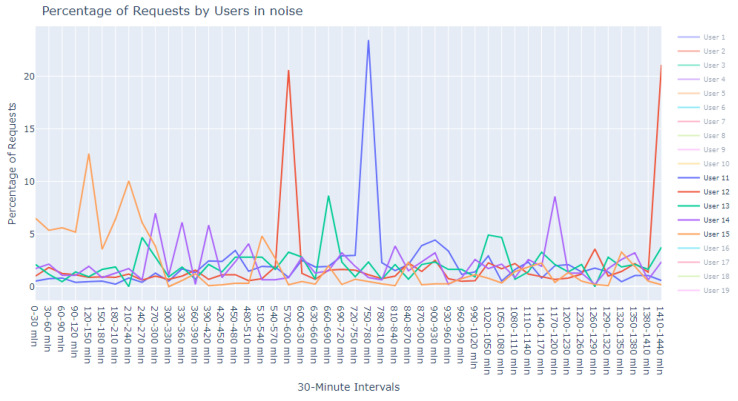
Anomalous noise users, user 11 to 15.

**Figure 19:**
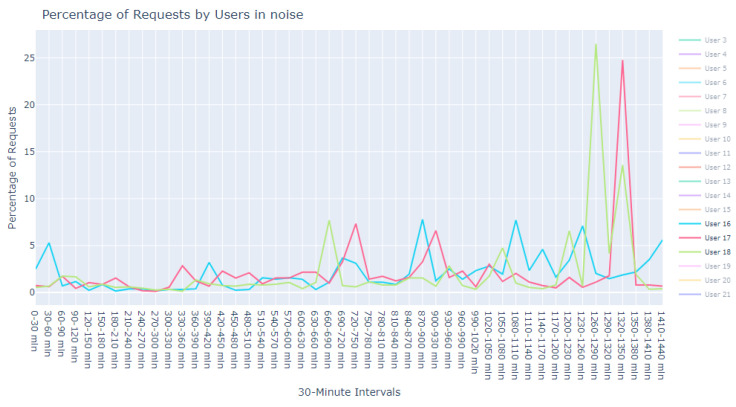
Anomalous noise users, user 16 to 18.

**Figure 20:**
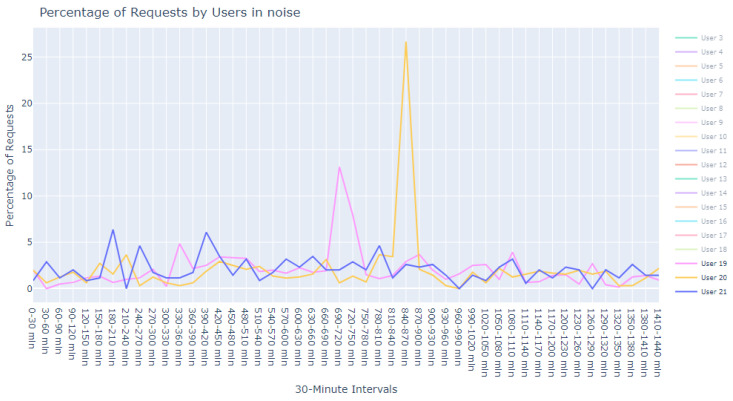
Anomalous noise users, user 19 to 21.

## 5. Results and Discussion

Our investigation into an enterprise network dataset through the application of an Isolation Forest algorithm, among other established methods, has yielded a powerful tool for the detection of anomalies amongst user behaviors. While traditional methods of anomaly detection often rely on prolonged monitoring and iterative expert analysis, which can necessitate multiple iterations to establish an abnormality benchmark, our method streamlines this process. It leverages the unique capabilities of isolation forests to effectively and efficiently identify outliers.

Our research introduced two distinctive strategies within this established framework. The first strategy focuses on pinpointing general anomalies across the dataset, while the second strategy delves into a more detailed inspection of individual user request patterns. This dual approach not only highlights widespread anomalies but also facilitates a deeper understanding of peculiarities in user request behaviors. It is instrumental in revealing not only anomalies within a day’s worth of traffic but also in establishing threshold levels for all examined features. This comprehensive scrutiny is essential for pinpointing key performance indicators, solidifying the importance of this novel framework for ongoing and future evaluations.

Our study presents a novel methodological framework for rapid anomaly detection within an enterprise network context. By using isolation forests, among other established methods, we dramatically streamline the identification of aberrant user behaviors. This approach equips security professionals with an effective and expedited means to explore their data, identifying potential security threats with newfound agility. The innovation lies in the rapid uncovering and analysis of unusual patterns, enabling a proactive stance in cybersecurity defense.

We believe that our framework for anomaly detection can be adapted and reused in various contexts beyond enterprise networks. While our study focuses on cybersecurity within this specific domain, the principles and framework we have developed have the potential to be applied to other fields where anomaly detection is relevant.

In summary, our proposed framework stands out in the field of anomaly detection for its algorithmic efficiency and general applicability. The utilization of Isolation Forests, among other established methods, allows for the efficient handling of large-scale data, while our unique feature set provides a more detailed analysis of user behavior. The validation process, incorporating both statistical analysis and expert evaluation, ensures the practical applicability of our findings. Collectively, these aspects underscore our framework’s comparative advantage in detecting sophisticated cyber threats in a rapidly evolving digital landscape.

### Limitations of the Current Approach

While our methodology demonstrates significant advancements in anomaly detection, there are inherent limitations in the current approach that warrant discussion:

1. Non-Real-Time Analysis: Our current model is not designed for real-time anomaly detection. This limitation may lead to delays in recognizing and addressing emergent threats, as the analysis is conducted on pre-collected datasets. The temporal gap between data collection and analysis could be critical in scenarios where immediate response is necessary.
2. Dependence on Historical Data: The effectiveness of our Isolation Forest algorithm is contingent upon the availability of historical data. In scenarios with limited or no prior data, such as newly deployed systems or applications, the model may face challenges in accurately identifying anomalies.
3. Adaptability to Evolving Threats: The current methodology may not be fully adaptable to rapidly evolving cyber threats. As attackers continually modify their strategies, static models may struggle to keep pace without frequent retraining and updates. This necessitates ongoing monitoring and model adjustments to maintain effectiveness against new types of anomalies.
4. Geographical Variance: Our approach does not currently account for the geographical dispersion of users, which can impact request times and patterns due to time zone differences. This presents a challenge in distinguishing between genuine anomalies and variations that are simply due to geographical factors.
5. Resource Utilization: The model’s resource efficiency, particularly in terms of CPU usage, is a key consideration. Optimizing the model for lower resource consumption is essential for enabling more frequent retraining cycles and handling larger datasets over extended periods.
6. False Positives and Negatives: While the model shows promise in detecting anomalies, there is a risk of false positives and negatives, which could lead to unnecessary alerts or overlooked threats. Future work will focus on refining the model’s accuracy by analyzing these occurrences in a meticulously labeled dataset.

In conclusion, recognizing these limitations is crucial for setting realistic expectations for the current model’s capabilities and for guiding future improvements. Our ongoing research aims to address these challenges, thereby enhancing the robustness and applicability of our anomaly detection.

## 6. Future Work

Looking ahead, our objective is to evolve this study into a dynamic framework that can perform real-time anomaly detection of user behavior. Building upon our initial findings, we aim to enhance the sophistication of our analyses by clustering various user anomalies. This will pave the way to not only recognize but also understand the intricate web of user interactions, leading to the creation of a meticulously labeled dataset. This dataset will be instrumental in evaluating the precision of our model by scrutinizing false positives and negatives, thus refining its accuracy.

A critical aspect of our future research will address the challenge posed by the geographical dispersion of users. The variance in request times due to differing time zones presents a unique obstacle that must be mitigated. Additionally, efforts will be made to optimize the model’s CPU usage, facilitating more frequent retraining cycles and enabling it to swiftly pinpoint anomalies across more extensive datasets, covering not just days but potentially weeks of user activity.

Furthermore, our future research will include a thorough comparative analysis. We aim to craft a robust benchmarking protocol to contextualize our method against existing literature and alternative approaches. This will ensure that our methodology not only stands on solid empirical ground but also contributes to the broader discourse on anomaly detection in enterprise networks. The study’s replicability and potential adaptation to different network setups will also be explored extensively in future research.

## Figures and Tables

**Table 1:** Metrics of studied approaches on experimental datasets [11].

Approach	PCA			LogCluster			IM			DeepLog			LogAnomaly		
**Dataset**	**P**	**R**	**F1**	**P**	**R**	**F1**	**P**	**R**	**F1**	**P**	**R**	**F1**	**P**	**R**	**F1**
D1	0.40	1.00	0.57	0.38	1.00	0.55	0.51	1.00	**0.68**	0.64	0.33	0.44	0.45	0.67	0.54
D2	0.56	0.43	0.48	0.50	0.57	0.53	0.34	0.43	0.38	0.71	0.86	**0.78**	0.71	0.86	0.78
D3	0.67	0.50	0.57	0.72	0.50	0.59	0.43	1.00	**0.60**	0.43	0.50	0.46	0.62	0.50	0.55
D4	0.20	0.44	0.28	0.24	0.53	**0.33**	0.32	0.21	0.26	0.17	0.38	0.23	0.22	0.34	0.27
D5	0.92	1.00	**0.96**	0.80	1.00	0.89	0.87	1.00	0.93	0.91	1.00	0.95	0.91	1.00	0.95
D6	0.64	1.00	0.78	0.86	0.81	0.83	0.82	1.00	0.90	0.93	0.96	0.94	0.92	1.00	**0.96**
D7	0.42	0.50	0.46	0.44	0.50	0.47	0.58	0.25	0.35	0.57	1.00	**0.73**	0.53	1.00	
D8	0.10	0.25	0.14	0.35	0.25	0.29	0.14	0.25	0.18	1.00	0.50	**0.66**	1.00	0.50	**0.66**
D9	0.18	0.60	0.28	0.28	1.00	0.44	0.44	0.40	0.42	0.32	1.00	0.48	0.54	0.60	**0.57**
D10	0.29	0.33	0.31	0.43	0.33	0.37	0.29	1.00	0.45	0.74	0.50	0.60	1.00	0.50	**0.67**
**Mean F1 (std)**	0.48 (0.24)			0.53 (0.19)			0.52 (0.24)			0.63 (0.22)			0.66 (0.19)		

**Table 2:** Dataset sample (hardware fingerprints were masked due to privacy).

Hardware_Fingerprint	Get_Count	Head_Count	Country	System	Status_200	...	Status_304	0	...	47
XXXXXX...0	3	3	en_us	Windows	3	...	0	0	...	0.00
XXXXXX...1	2	2	es_es	Windows	0	...	2	0	...	0.00
XXXXXX...2	1	1	pl_pl	Windows	1	...	0	0	...	2
XXXXXX...3	5	425	en_us	Windows	10	...	420	0	...	12

**Table 3:** Descriptive statistics for threshold identification.

	Get_Count	Head_Count	Status_200	Status_401	Status_304	Status_404	Status_416	Status_206	0	...	47
**mean**	108.69	137.17	36.93	114.10	94.62	0.07	0.00	0.14	3.66	...	2.82
**std**	977.59	766.55	544.45	950.12	580.02	3.09	0.00	3.98	25.67	...	28.78
**min**	0.00	0.00	0.00	0.00	0.00	0.00	0.00	0.00	0.00	...	0.00
**25%**	6.00	29.00	4.00	1.00	7.00	0.00	0.00	0.00	0.00	...	0.00
**50%**	25.00	60.00	9.00	18.00	38.00	0.00	0.00	0.00	0.00	...	0.00
**75%**	104.00	143.00	21.00	110.00	92.00	0.00	0.00	0.00	2.00	...	2.00
**max**	160,662.00	163,096	90,439.00	183,238.00	100,365.00	384.00	1.00	608.00	3106.00	...	4301.00

**Table 4:** Anomalous users sample.

	Get_Count	Head_Count	Country	System	Status_200	Status_401	Status_304	Status_404	Status_416	Status_206	0	...	47
**user0**	160,662	163,096	he_il	Windows	90,439	183,238	49,847	55	0	175	3106	...	3492
**user1**	63,246	0	tr_tr	Android	63,245	0	0	0	0	0	169	...	0
**user2**	100,377	0	tr_tr	Android	12	0	100,365	0	0	0	0	...	50
**user3**	60,437	0	pl_pl	Android	60,437	0	0	0	0	0	0	...	0

**Table 5:** Update behaviour dataset sample.

	0	1	2	3	...	47
**user1**	2%	2%	3%	1%	...	0%
**user2**	2%	2%	2%	2%	...	5%
**user3**	4%	3%	2%	3%	...	3%
**user4**	1%	3%	2%	1%	...	1%
**user5**	3%	3%	4%	1%	...	1%

## Data Availability

Third Party Data Restrictions apply to the availability of these data. Data were obtained from ESET, spol. s r.o. and are available from the authors with the permission of ESET, spol. s r.o.

## Abbreviations

The following abbreviations are used in this manuscript:

DBSCAN	Density-Based Spatial Clustering of Applications with Noise
ERDF	European Regional Development Fund
GDPR	General Data Protection Regulation
IDS	Intrusion Detection Systems
IM	Invariant Mining
K-NN	K-Nearest Neighbors
LSTM	Long Short-Term Memory
MACCDC	Mid-Atlantic Collegiate Cyber Defense Competition
PCA	Principal Component Analysis
t-SNE	t-Distributed Stochastic Neighbor Embedding
